# Effect of antenatal colostrum expression teaching on breastfeeding practices and birth outcomes: a retrospective case-control chart review

**DOI:** 10.1186/s12884-026-09298-5

**Published:** 2026-05-26

**Authors:** Claire Niessen, Naida Hawkins, Tin-Wing Yen, Kelsey M Cochrane

**Affiliations:** 1 College of Pharmacy and Nutrition, Division of Nutrition, The University of Saskatchewan, 107 Wiggins Rd, Saskatoon, SK S7N 5E5 Canada; 2 More Milk Sooner, North Battleford, SK Canada; 3 College of Medicine, Department of Obstetrics and Gynecology, The University of Saskatchewan, Saskatoon, SK Canada

**Keywords:** Breastfeeding, Antenatal colostrum expression, Human milk, Obstetrics, Maternal and infant health

## Abstract

**Background:**

Antenatal colostrum expression is a practice involving self-massage of the breasts starting in late pregnancy (typically 35-36-weeks of gestation), which is thought to stimulate lactation and allow individuals to secrete and store colostrum prior to delivery. To support successful breastfeeding initiation and continuation among patients, some clinicians provide antenatal colostrum expression teaching as part of standard prenatal care. However, the impact of antenatal colostrum expression teaching is unclear due to limited evidence which is largely anecdotal. The primary aim of this case-control chart review was to evaluate the effect of antenatal colostrum expression teaching as part of standard obstetrical care on breastfeeding practices at first postpartum checkups, gaining pilot data to inform future research. Secondary outcomes included the evaluation of preterm delivery, mode of delivery, and repeat hyperbilirubinemia screening between groups.

**Methods:**

A retrospective case-control chart review before (*n* = 79) and after (*n* = 96) antenatal colostrum expression teaching was incorporated into standard care at 36-gestational week visits in an obstetrical practice in North Battleford, SK, Canada (2016–2020). Odds of fully breastfeeding at first postpartum check-ups in cases vs. controls was evaluated with logistic regression, adjusted for parity, mode of delivery, and infant age at postpartum check-ups. Odds of repeat hyperbilirubinemia screening in cases vs. controls was evaluated with logistic regression, adjusted for parity, mode of delivery, and infant sex.

**Results:**

Antenatal colostrum expression teaching increased the odds of fully breastfeeding at postpartum check-ups by > 3-fold as compared to controls (aOR = 3.09; 95% CI = 1.35–7.09). Odds of repeat hyperbilirubinemia screening was 59% lower in cases vs. controls (aOR = 0.41; 95% CI = 0.18–0.92). No significant differences in gestational age at delivery or mode of delivery were found between groups.

**Conclusion:**

Antenatal colostrum expression presents a promising intervention to help prepare patients mentally and physiologically for breastfeeding and labour. While these findings suggest that advocacy efforts are warranted to increase standard provision of antenatal colostrum expression teaching in late gestation, results should be confirmed in a randomized intervention trial, which further aims to understand the effect on longer-term breastfeeding rates and infant health.

## Background

Exclusive breastfeeding for the first 6 months of life is universally recommended by the American Academy of Pediatrics, Canadian Paediatric Society, and World Health Organization to support optimal maternal and infant health and development [1–3]. This is supported by a large body of evidence for the role of breastfeeding in supporting both short- and long-term health outcomes, such as programming the immune system and preventing infections [4–9], improving neurocognitive development [10–12], and reducing chronic disease risk [13–17], and providing protection against breast and ovarian cancer in the lactating individual [18, 19]. Despite being the normal and natural way to infant feed, many obstacles may impact an individual’s ability to establish breastfeeding; this may include delayed secretory activation in the mammary gland, resulting in insufficient milk production following delivery [20]. Delayed breastfeeding may have serious consequences for infant health (due to possible slow weight gain, failure to thrive, dehydration, etc.) and contributes to maternal stress, ultimately reducing the likelihood of continued breastfeeding [21–23].

Antenatal colostrum expression is a practice involving self-massage of the breasts, which aims to improve breastfeeding initiation and continued postnatal breastfeeding success [24]. Universal standards of practice for antenatal colostrum expression do not exist, however, individuals may be instructed to begin hand massage in late pregnancy (~ 35–36 weeks of gestation), starting with once daily until 37 weeks, followed by multiple times per day (as desired) until delivery [25]. While the effect of antenatal colostrum expression on breastfeeding success remains inconclusive [24], previous research suggests that it may reduce the time to establish sufficient milk flow, decrease the early introduction of infant formula in hospital, and increase maternal confidence in their ability to produce sufficient milk and continue breastfeeding after hospital discharge [26–30]. However, some report negative experiences, including stress around breastfeeding if they are unable to express colostrum antenatally or concerns that antenatal colostrum expression may reduce nutrition available for the fetus [31]. While the physiology underpinning the effects of antenatal colostrum expression on breastfeeding initiation remain unclear, proposed hypotheses include increased oxytocin release following stimulation of the areola and nipple areas [32], and stimulation of secretory activation due to early colostrum removal [33].

Potential benefits following antenatal colostrum expression for neonatal health may be related to the provision of more colostrum to the neonate sooner [34]; this is particularly true if breastfeeding initiation challenges arise, as colostrum may be collected and stored prenatally for postpartum hospital use. Timely and adequate feeding after delivery helps the infant to more rapidly pass meconium, the bilirubin-rich first stool [35, 36]. For infants born to those with gestational diabetes mellitus, antenatal colostrum expression may enable the provision of colostrum, as opposed to infant formula, for hypoglycemia prevention [37, 38]. While it is unclear whether colostrum is more effective than formula in reducing neonatal hypoglycemia [30, 39, 40], minimizing formula introduction in hospital may support breastfeeding exclusivity and longevity after hospital discharge [41].

Antenatal colostrum expression may further contribute to natural labor induction, as breast stimulation causes the uterus to contract (possibly due to increased oxytocin release) [42, 43], and increases cervical ripening (per Bishop Score) [44]. Per a 2005 Cochrane Systematic Review of clinical trials including those due for third trimester induction of labour, breast stimulation (as compared to no intervention) was associated with a reduced number of individuals still in labour after 72 h (62.7% versus 93.6%; RR: 0.67; 95% CI 0.6 to 0.74) [42]; however, mode of delivery (vaginal vs. caesarean section) does not appear to be meaningfully impacted [34, 42]. In a 2023 clinical trial (*n* = 304), odds of spontaneous labor was ~ 2-fold higher (OR: 2.09; 95% CI: 1.05–4.14) in those randomized to antenatal colostrum expression counselling vs. controls who received standard education [34]. Thus, contraindications for antenatal colostrum expression in those with placental abnormalities or planned caesarean deliveries has been suggested by some as a conservative approach to reduce the risk of early labour [42]. Whether this precaution is warranted is unclear, as other studies (representing > 900 births) have reported no difference in gestational weeks at delivery or preterm delivery due to antenatal colostrum expression [30, 39].

The primary aim of this retrospective case-control study was to evaluate breastfeeding practices at first postpartum checkups among mother-infant pairs before and after antenatal colostrum expression teaching was incorporated into standard obstetrical care in North Battleford, SK, Canada, gaining pilot data to inform future research to confirm any effects found. Secondary outcomes included rates of preterm delivery, mode of delivery, repeat hyperbilirubinemia screening, and birth weight regain at postpartum checkups.

## Methods

A retrospective case-control chart review was conducted using electronic medical records (EMR) in the Saskatchewan Health Authority MedAccess EMR (former Prairie North Health Region). Standardized provision of face-to-face antenatal colostrum expression teaching in late pregnancy was introduced in April 2018 in the obstetrical practice of Dr. Yen in North Battleford, SK, Canada (during 36 gestational week prenatal appointments; coinciding with standardized group B streptococcus rectovaginal swab collections). Controls were considered pregnant patients seen for 36-week visits from April 2016 to March 2018, and cases were considered those seen for 36-week visits after April 2018 to December 2019. A cut-off date of December 2019 was chosen to reduce the influence of the Covid-19 pandemic in early 2020 and associated disruptions to obstetrical and standard health care practices, while maintaining similar total timeframes and sample sizes for cases and controls. Per Saskatchewan Health Authority standards, the typical length of stay following a vaginal delivery is 24–36 h, and 48 h following a Caesarean Section delivery [45]. Postpartum well-baby visits are offered to all parents in Saskatchewan at approximately day 7–10 postpartum, and are conducted at patient homes by a public health nurse [46]. Exclusion criteria included high-risk pregnancies, resulting in transfer of care prior to 36 gestational weeks, multiple pregnancies, pregnancy loss, stillbirth, or a Personalized Health Number (PHN) outside of Saskatchewan. A waiver of consent was obtained from the University of Saskatchewan Biomedical research ethics board (UofS-4806) to protect patient confidentiality. This study was approved by the University of Saskatchewan Biomedical research ethics board (UofS-4806), in accordance with the Declaration of Helsinki and received Saskatchewan Health Authority operational approval for electronic chart access (OA-UofS-4806).

Those who received antenatal colostrum expression teaching were given a hand expression kit (created by More Milk Sooner, a clinician-led Saskatchewan-based organization) and face-to-face teaching from the obstetrician during their prenatal appointment. Physician education provided to all cases included verbal recommendations to begin self-massage of the breasts immediately, starting once per day until 37 gestational weeks, followed by multiple times per day until delivery, with written instructions to hand express once daily x 1 week for 5 min (starting at ~ 36 gestational weeks), and then 3–4 times daily x 5–10 min for the remainder of pregnancy. Kits included 3 × 1 mL sterile colostrum collectors (syringes) and a handout with a link to the More Milk Sooner website (www.moremilksooner.com/), which has numerous other educational resources to support antenatal colostrum expression and breastfeeding more generally.

### Data abstraction

Maternal Saskatchewan PHNs for eligible participants from April 2016-December 2019 were identified in Dr. Yen’s obstetrical clinic patient records. Maternal PHNs were subsequently identified in the Saskatchewan Health Authority MedAccess EMR, and used to locate associated maternal hospital intake forms, infant PHNs, infant birth intake forms, and public health nurse chart notes for first postpartum check-ups. Data, as available, abstracted from all sources included maternal age, infant sex, parity, gestational weeks at delivery, preterm birth (< 37 gestational weeks), mode of delivery, postpartum hemorrhage, maternal transfusion, infant transfer to the neonatal intensive care unit prior to hospital discharge, birth weight (g), discharge weight (g), repeat hyperbilirubinemia screening (based on initial screening indicating an elevated transcutaneous bilirubinometry), infant feeding at discharge. Data from postpartum check-ups included infant age (days), weight (g), whether birth weight was re-gained (restricted to those ≥ 10 days postpartum), and infant feeding. De-identified data was recorded in REDCap (University of Saskatchewan). Infant feeding practices were classified as fully breastfeeding, partial breastfeeding, and no breastfeeding, reflecting how the infant was fed at each timepoint of interest.

### Sample size considerations

This study aimed to gain pilot data and to explore the effect of antenatal colostrum expression teaching (provision of More Milk Sooner kits and obstetrician-led teaching) on perinatal outcomes of interest (breastfeeding practices, infant health outcomes including possible hyperbilirubinemia). The largest sample size possible was achieved by including all eligible patients during the identified date ranges. These data can be used to guide next steps for research and advocacy efforts, and may inform an adequately powered intervention trial to evaluate the effect of antenatal colostrum expression teaching vs. none in late gestation.

### Statistical analyses

Data were summarized descriptively using a mean ± SD (or median, IQR, if not normally distributed) and counts with percentages. Maternal and infant characteristics, birth outcomes, and infant feeding in cases vs. controls were compared with a two-sample t-test or chi-squared test (or nonparametric equivalent, as appropriate). Odds of fully breastfeeding at first postpartum check-ups in those who received antenatal colostrum expression teaching (cases) vs. those who did not (controls) was evaluated with logistic regression, adjusted for parity, mode of delivery, and infant age at the postpartum check-up. Odds of repeat screening for hyperbilirubinemia after birth in those who received antenatal colostrum expression teaching (cases) vs. those who did not (controls) was evaluated with logistic regression, adjusted for parity, mode of delivery, and infant sex. All analyses were completed on an intention-to-treat basis with no imputation for missing data.

## Results

**Fig. 1:**
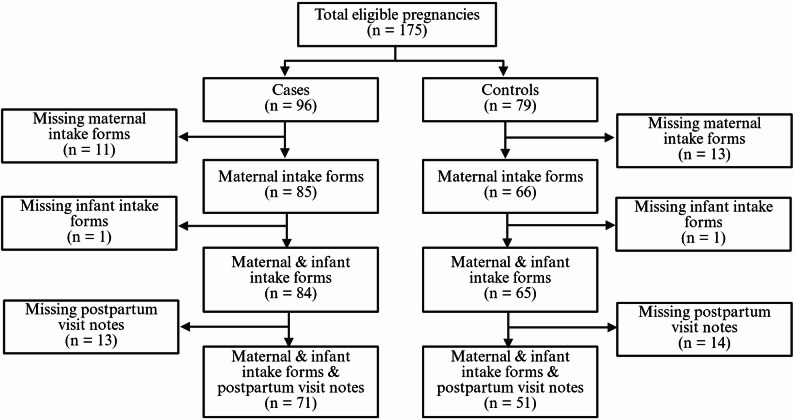
Participant flow chart

A total of 175 eligible pregnancies were identified from Dr. Yen’s patient records, including *n* = 79 controls (Apr 2016-Mar 2018) and *n* = 96 cases (Apr 2018-Dec 2019). Corresponding maternal and infant hospital intake forms from the Battlefords Union Hospital were available in the Saskatchewan Health Authority EMR for *n* = 84 cases (*n* = 11 missing maternal forms; *n* = 1 missing infant form) and *n* = 65 controls (*n* = 13 missing maternal form; *n* = 1 missing infant form). Public health nurse chart notes for postpartum infant check-ups were available for *n* = 71 cases and *n* = 51 controls. See Fig. 1 for participant flow chart.

Maternal and infant characteristics, birth outcomes, and infant feeding are reported in Table 1. Characteristics of cases and controls appeared similar in most regards; only infant sex varied significantly between groups, with more female babies born in cases (60%) vs. controls (36%). Participants were on average 29 years of age. Mean gestational weeks at delivery was ~ 38 weeks overall, with *n* = 2 infants in both groups delivering prematurely (< 37 gestational weeks). Possible hyperbilirubinemia (based on repeat screening required) was lower among cases (25%) vs. controls (40%). Rates of caesarean section delivery were higher among cases (39%) vs. controls (24%), with lower rates of fully breastfeeding at hospital discharge among cases (38%) vs. controls (52%). Postpartum check-ups overall occurred at a median of day 9 postpartum (interquartile range = day 5 to day 14). At postpartum check-ups, rates of fully breastfeeding were significantly higher among cases (51%) as compared to controls (27%). Infant birth weight was similar between groups (median weight 3369–3395 g overall), with a higher proportion of infants who regained their birth weight among cases (94%) vs. controls (86%).

**Table 1:** Maternal and infant characteristics, birth outcomes, and infant feeding among cases vs. controls

	*n*	Cases	*n*	Controls	*P*
Maternal age, years (mean, SD)	85	29 (5)	66	29 (5)	^b^0.811
Nulliparous, yes	85	40 (47%)	66	28 (42%)	^c^0.570
Gestational weeks at delivery (mean, SD)	84	38.9 (1.1)	64	38.6 (1.1)	^b^0.298
Preterm delivery (< 37 weeks), yes	84	2 (2%)	64	2 (3%)	^d^1.00
Mode of delivery	84		66		^c^0.051
Vaginal		51 (61%)		50 (76%)	
Caesarean section		33 (39%)		16 (24%)	
Postpartum hemorrhage, yes	71	3 (4%)	51	2 (4%)	^d^1.00
Maternal postpartum transfusion, yes	67	0 (0%)	48	2 (4%)	^d^0.172
Infant transfer to NICU, yes	85	4 (5%)	62	0 (0%)	^d^0.138
Birth weight (median, IQR)	84	3395 (3123, 3575)	65	3395 (3120, 3690)	^e^0.879
Infant sex, female	84	50 (60%)	66	24 (36%)	^c^0.005
Repeat hyperbilirubinemia screening, yes	77	19 (25%)	58	23 (40%)	^c^0.063
Infant feeding at discharge	77		60		^d^0.237
Fully breastfeeding		29 (38%)		31 (52%)	
Partial breastfeeding		41 (53%)		26 (43%)	
No breastfeeding		7 (9%)		3 (5%)	
Postpartum infant age (median, IQR)	71	10 (5, 15)	51	8 (6, 12)	^e^0.484
Postpartum infant feeding	71		51		^d^0.023
Fully breastfeeding		36 (51%)		14 (27%)	
Partial breastfeeding		29 (41%)		33 (65%)	
No breastfeeding		6 (8%)		4 (8%)	
Postpartum infant weight (median, IQR)	65	3430 (3180, 3740)	50	3355 (3090, 3760)	^e^0.443
^a^Birth weight re-gained, yes	36	34 (94%)	21	18 (86%)	^d^0.346

^a^Restricted to those ≥ 10 days postpartum

Statistical test used: ^b^two-sample t-test; ^c^Chi-squared test; ^d^Fisher’s exact test; ^e^Wilcoxon rank sum test

The unadjusted and adjusted odds of repeat hyperbilirubinemia screening and fully breastfeeding at postpartum check-ups in those who received antenatal colostrum expression teaching (cases) vs. those who did not (controls) is presented in Table 2. Odds of repeat hyperbilirubinemia screening was 59% lower in cases vs. controls (aOR = 0.41; 95% CI = 0.18–0.92), adjusted for parity, mode of delivery, and infant sex. Further, antenatal colostrum expression teaching significantly increased the odds of fully breastfeeding at postpartum check-ups by over 3-fold as compared to controls (aOR = 3.09; 95% CI = 1.35–7.09), adjusted for parity, mode of delivery, and infant age (days postpartum).

**Table 2:** Odds of fully breastfeeding at postpartum check-ups and repeat hyperbilirubinemia screening among cases vs. controls

Odds of fully breastfeeding at first postpartum check-ups				
	Odds ratio	95% CI	^a^Adjusted odds ratio	95% CI
Participants (*n*)	122	*--*	121	*--*
Antenatal colostrum expression teaching				
Yes	2.72	1.23 to 5.88	3.09	1.35 to 7.09
Parity	--	--		
Multiparous	--	--	0.33	0.15 to 0.75
Mode of delivery	--	--		
Vaginal	--	--	3.07	1.27 to 7.39
Infant age (days)	--	--	0.99	0.91 to 1.07

Odds of repeat hyperbilirubinemia screening				
	Odds ratio	95% CI	^b^Adjusted odds ratio	95% CI
Participants (*n*)	135	*--*	131	*--*
Antenatal colostrum expression teaching				
Yes	0.50	0.24 to 1.04	0.41	0.18 to 0.92
Parity	--	--		
Multiparous	--	--	0.78	0.36 to 1.69
Mode of delivery	--	--		
Vaginal	--	--	2.25	0.94 to 5.39
Infant sex	--	--		
Male	--	--	0.55	0.25 to 1.23

^a^Adjusted for parity, mode of delivery, and infant age at postpartum check-up

^b^Adjusted for parity, mode of delivery, and infant sex

## Discussion

This investigation provides promising and novel data regarding the potential benefits of antenatal colostrum expression teaching for improving breastfeeding rates and birth outcomes. The most staggering finding was that those who were given antenatal colostrum expression teaching were > 3 times more likely to fully breastfeed after discharge (95% CI: 1.35 to 7.09). However, infant feeding at hospital discharge was not statistically different between groups, with lower rates of fully breastfeeding among cases. We propose that differences in mode of delivery may underscore this finding, given a higher rate of caesarean section delivery among cases, which is associated with formula supplementation in hospital. As cases were more likely to fully breastfeed by first postpartum check-ups, we hypothesize that the provision of infant formula in hospital may be less detrimental to transitioning back to fully breastfeeding in those who practice antenatal colostrum expression. This may be due to increased maternal confidence to support continued breastfeeding following antenatal hand expression. Further, early colostrum removal may improve milk establishment through stimulation of secretory activation (similar to earlier milk removal following delivery [47, 48]), potentially leading to better ongoing milk supply and breastfeeding rates.

Higher formula provision in hospital among cases is additionally surprising as theoretically, cases may have brought antenatal colostrum to the hospital which could have been used in addition to or in place of formula if a supplement was required. Whether cases in this study had antenatal colostrum available is unknown, as this data was not recorded in maternal and infant intake forms. Following Baby Friendly Initiative [49] standards, formula introduction in hospital should be avoided when possible, as it is associated with reduced total exclusive breastfeeding duration (per the World Health Organization definition of exclusive breastfeeding [50]) as reported by the multi-province Canadian CHILD cohort (*n* = 3195) [41]. While results from the current study suggest that the impact of formula introduction in hospital on continued breastfeeding may be partially mitigated by antenatal colostrum expression teaching, breastfeeding exclusivity would be most effectively supported by hospital practices which reduce any unnecessary formula provision in the first place, such as protocols for the safe use of antenatal colostrum.

Most previous studies report increased breastfeeding initiation and reduced formula provision in hospital in those who practice antenatal colostrum expression [27, 30, 34, 39]. In a case-control prospective study in India, time to initiate lactation was evaluated in *n* = 100 pregnant individuals selected to practice breast massage starting at 37 gestational weeks vs. a comparator group of *n* = 100 [27]; a significantly higher number of cases as compared to controls were able to achieve full lactation within 6 h (89% vs. 72%) [27]. In a multicenter randomized controlled trial in Australia, pregnant individuals with gestational diabetes were randomized to antenatal colostrum expression (*n* = 319) or standard care (*n* = 316) [39]; breastfeeding exclusivity was significantly higher 24 h after delivery following antenatal colostrum expression (57% vs. 49%; RR = 1.15; 95% CI: 1.03–1.30) [39]. Data on long-term breastfeeding rates following antenatal colostrum expression remains less compelling, with previous studies reporting no significant differences after 3 months [39] and 6 months [34, 50].

The requirement for increased bilirubin screening has important clinical implications, as additional testing contributes to delays in hospital discharge, increased financial strain on the health care system, and stress among new parents. While hyperbilirubinemia occurs commonly among healthy-term infants and is typically non-pathological, there is a risk of progression to acute bilirubin encephalopathy, causing severe, irreversible neurological damage [51, 52]. In this study, antenatal colostrum expression teaching was associated with a 59% reduction in odds of repeat bilirubin screening (indicative of possible hyperbilirubinemia), adjusting for parity, mode of delivery, and infant sex (aOR = 0.41; 95% CI: 0.18–0.92). However, this finding should be interpreted with caution. Theoretically, the provision of more colostrum sooner, due to rapid lactation initiation or the ability to provide stored colostrum, may contribute to more rapid passing of the bilirubin-rich meconium; however, this chart review did not have access to whether antenatally-expressed colostrum was provided among cases, thus underpinning the need for further investigation. It is also possible that this finding is indicative of increased formula provision in hospital among cases, as rapid feeding in general (within 1 h of delivery) helps the infant to pass meconium [35, 36]. Of note, while breastfeeding in general is commonly considered a risk factor for elevated bilirubin (known as “breastfeeding jaundice”), this is almost always related to suboptimal intake and excess weight loss, rather than breastfeeding per se [52]. As antenatal colostrum expression may stimulate lactation, theoretically making higher volumes of milk available sooner, future research should also evaluate its effect on rates of “breastfeeding jaundice”. Ultimately, rapid feeding with continued, effective breastfeeding, and early bilirubin screening are the most effective strategies for hyperbilirubinemia prevention [35, 36, 51, 53].

Other birth outcomes explored included gestational age at delivery and mode of delivery. As is supported by other studies [34, 39, 54], we found no difference in rates of preterm delivery between those taught antenatal colostrum expression vs. controls. This provides further evidence that contraindications to antenatal colostrum expression which aim to reduce the risk of early labour may not be warranted. While rates of caesarean section delivery were higher among cases (39%) vs. controls (24%), we do not believe that this was due to antenatal colostrum expression. Risk factors for caesarean delivery include placental abnormalities (e.g., placenta previa, accreta, or abruption), compressed umbilical cord, fetal positioning (breech presentation) or size (very large babies), certain maternal medical conditions (e.g., infectious diseases, cervical cancer, cardiac or pulmonary disease), and most importantly, a previous caesarean delivery [55–57]; we were not able to evaluate most of these risk factors in the current study. Finally, other studies do not support an association of antenatal colostrum expression with increased risk of caesarean delivery [30, 34, 39, 42, 54]. However, future studies should investigate the effect of antenatal colostrum expression on rates of artificial labour induction, given pilot data for increased cervical ripening (Bishop Score) [44]. In this way, antenatal colostrum expression may actually reduce the risk of caesarean delivery; however, a very large sample size would be required to demonstrate this association, with ability to control for known medical and physiological risk factors.

Other outcomes of interest for future research include colostrum composition before and after delivery (including nutritional and immunological components) and how the practice of antenatal colostrum expression impacts overall milk volume and time to establish a full milk supply; this could be impactful for combatting the top parent-reported reason for breastfeeding cessation prior to 6 months in Canada, a perceived insufficient milk supply [58].

Strengths and limitations of the current study are discussed below. Strengths include the use of one obstetrician for all study participants, helping to eliminate potential bias of physician-to-physician differences in antenatal colostrum expression teaching approach, or in general care prior to standardized teaching. Further, More Milk Sooner kits were carefully designed by pregnancy and lactation experts with input from pregnant individuals and parents during kit design and resource development to ensure an effective, high-quality teaching tool which is clear and easy to follow. Of note, all forms of potential harm (e.g., maternal mental health [31]) should be considered when advising antenatal colostrum expression, with counseling strategies that aim to reduce pressure to produce a certain amount of colostrum and normalizing a lack of any colostrum expressed despite practicing breast massage. While we were able to explore the effect of antenatal colostrum expression teaching on various birth outcomes, we did not have access to some pertinent data, such as whether or how antenatal colostrum expression was practiced and whether antenatal colostrum was brought to the hospital or given after delivery, bilirubin concentrations or confirmation of jaundice diagnosis, history of caesarean section delivery, or whether deliveries were artificially induced. There was a higher rate of loss-to-follow-up among controls (15%) vs. cases (8%) to assess infant feeding changes from delivery to postpartum visits; this may have inflated differences between groups. Finally, while we attempted to reduce variability via the use of one physician and by including a fairly tight total timeframe (April 2016-December 2019), it is possible that changes in hospital practice occurred over time or breastfeeding knowledge among participants evolved between cases and controls.

## Conclusion

Antenatal colostrum expression is a seemingly low-risk intervention that pregnant individuals can conduct themselves to prepare mentally and physiologically for breastfeeding and labour, providing an increased sense of agency in the birthing process [28]. However, teaching requires sensitivity and tact to avoid putting additional pressure on pregnant individuals [31]. This study aims to guide next steps and advocacy efforts for antenatal colostrum expression teaching and inform an adequately powered intervention trial to confirm effects on maternal and infant health; we suggest focusing on longer-term breastfeeding exclusivity (up to 6 months postpartum), infant jaundice, and artificial labour induction. While effects found in this study should be confirmed in an adequately powered trial, antenatal colostrum expression presents a promising intervention which may improve breastfeeding continuation after hospital discharge.

## Data Availability

The datasets analysed in this study are not publicly available but may be accessible from the corresponding author upon reasonable request.

## Abbreviations

EMR: Electronic medical records
PHN: Personalized health number
